# The impact of renal dysfunction after critical illness on the management of cancer

**DOI:** 10.3389/fneph.2025.1597253

**Published:** 2025-05-22

**Authors:** Thiago Gomes Romano, Rodrigo Chaves, Izabela Sinara Alves, Henrique Palomba

**Affiliations:** ^1^ General ICU, Vila Nova Star Hospital/Oncological and Neurological ICU, São Luiz Itaim Hospital/General ICU, São Luiz Alphaville Hospital/Discipline of Nephrology, ABC School of Medicine/Rede D’or Research and Teaching Institute, São Paulo, Brazil; ^2^ Discipline of Nephrology, ABC School of Medicine, São Paulo, Brazil; ^3^ Hospital São Luiz Alphaville/Cancer Institute of the State of São Paulo, São Paulo, Brazil; ^4^ General ICU, Vila Nova Star Hospital/Oncology and Neurological ICU, São Luiz Itaim Hospital/São Luiz Itaim and Vila Nova Star Hospital, São Paulo, Brazil

**Keywords:** acute kidney injury (AKI), post intensive care syndrome (PICS), cancer, chemotherapy, intensive care

## Abstract

A 67-year-old male patient with limited-stage diffuse large B-cell lymphoma was on an R-CHOP (rituximab, cyclophosphamide, doxorubicin, vincristine and prednisone) chemotherapy regimen. His Eastern Cooperative Oncology Group (ECOG) Performance Scale score was zero, indicating functional independence for activities of daily living. The patient was admitted to the intensive care unit (ICU) with septic shock in the presence of febrile neutropenia progressing to acute kidney injury, hypoxemic respiratory failure, and systemic arterial hypotension, in addition to the already established hematological dysfunction with thrombocytopenia. During his 32-day ICU stay, he required invasive mechanical ventilation, renal replacement therapy (RRT) and vasopressor drugs, with a focus on control of the infection. The patient was discharged from the ICU with sarcopenia and a serum creatinine level of 2.3 mg/dL, indicating a clearance rate of 24 ml/min/1.73 m^2^. Oxygen supplementation was needed. What impact did critical illness, more specifically renal dysfunction, have on the planning of onco-hematological treatment in this patient?

## Introduction

Currently, approximately 15% of beds in intensive care units (ICUs) are occupied by cancer patients (1), with 5% of solid cancer patients and 15% of hematological patients being admitted to the ICU within 2 years after cancer diagnosis (2).

We also know that 22% of patients with newly diagnosed leukemia and 17% with lymphoma will need ICU treatment at some point in their disease course (3). Although recent treatment regimens, such as monoclonal antibodies for non-Hodgkin’s lymphoma, aggressive regimens for acute lymphoblastic leukemia and Burkitt’s lymphoma, and transretinoic acid for promyelocytic leukemia, promote improved survival and remission rates, the prolongation of the lives of these patients also increases their likelihood of ICU stay (4–6).

Recent medical literature has also shown that the prognostic factors of cancer in patients in the ICU are more strongly correlated with the condition of their critical illness, such as the need for mechanical ventilation, acute kidney injury (AKI), shock, and respiratory failure, than with their individual oncological characteristics (7).

**Figure 1:**
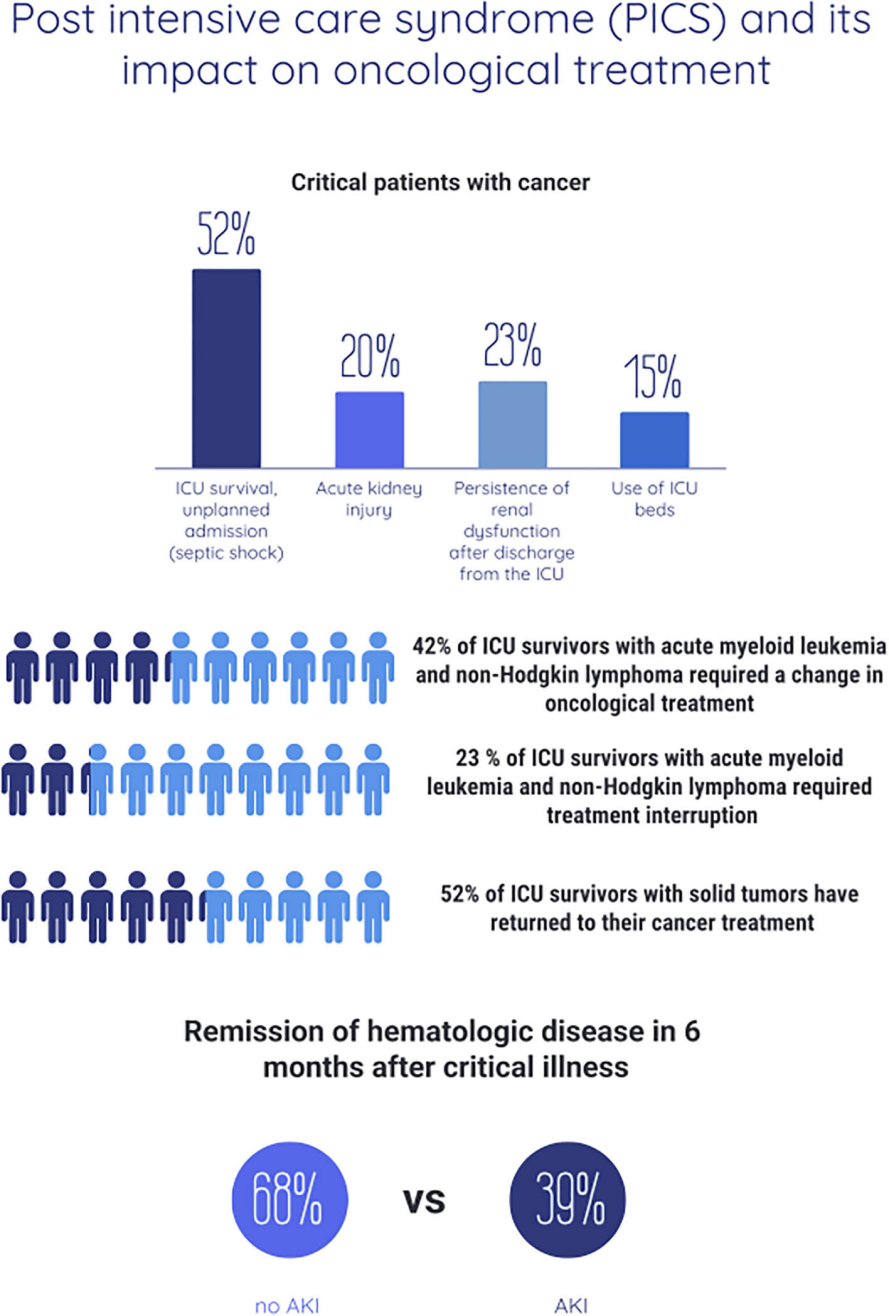
Known data on the impact of post-intensive care syndrome (PICS) on cancer management.

In addition to the high use of ICU resources and the knowledge of prognostic factors in this population, another remarkable finding is the decrease in mortality of the critically ill oncohematological cancer population over the last few decades, albeit with an impact on greater frailty, cognitive impairment and persistence of postdischarge organ dysfunctions (3, 8–10), the so-called post-intensive care syndrome (PICS) (11–13) (**Figure 1**).

Despite this shift in outcomes, few studies explore how cancer treatment is affected after ICU discharge, particularly in the context of organ dysfunction. In this narrative review, we explore the impact of PICS—focusing on persistent renal dysfunction after AKI—on the therapeutic management of cancer.

## Impact of critical illness on cancer therapeutic management

Up to 64% of ICU survivors may experience impairments in one or more of the three core domains: cognitive, physical, or psychiatric, within 3 months of discharge (14). Caregivers and family members (PICS-Family) are also at risk of psychological distress, including anxiety, depression, and post-traumatic stress.

Risk factors for PICS are widespread in ICU care and range from pre-existing conditions (e.g., baseline anxiety or depression) to ICU-acquired complications such as mechanical ventilation, delirium, sedation, glucose dysregulation, renal replacement therapy (RRT), transfusions, and sepsis (15–18).

Cognitive impairment affects up to 78% of ICU survivors (17, 19). The BRAIN-ICU study, which evaluated 821 patients prospectively, revealed that 40% of patients showed cognitive dysfunction at 3 months post-discharge. Among these, 26% had deficits similar to moderate traumatic brain injury or mild dementia (20). These impairments often affect daily activities and may persist long term (21).

In the psychiatric domain, epidemiological studies report PTSD, anxiety, and depression in 1–62% of ICU survivors, with some data suggesting an increased risk of suicide and self-harm (HR 1.22, 95% CI 1.11–1.33) compared with patients not admitted to the ICU (22).

In the physical domain, conditions such as sarcopenia, malnutrition, reduced pulmonary function, and persistent organ dysfunction may affect approximately 25% of ICU survivors (20). Among these, persistent renal dysfunction has emerged as a key factor in the post-ICU cancer population and is the central focus of this review, given its direct impact on chemotherapy management.

## Impact of renal dysfunction after ICU discharge on therapeutic management

Cancer patients represent a population at risk for the development of acute kidney injury (AKI), which can result from exposure to nephrotoxic drugs, infectious complications, tumor lysis, hypercalcemia and obstructive uropathy (23, 24). The annual incidence of AKI in noncritically ill cancer patients ranges from 11 to 20%, with a higher prevalence in the hematological population.

In patients who have undergone hematopoietic stem cell transplantation (HSCT) (25–27), even small elevations in creatinine levels are associated with increased mortality (28), and the need for RRT occurs in approximately 5% of patients; however, in severe cases, this percentage can range from 8 to 60% (29).

The long-term prognosis of renal recovery after an episode of AKI is an issue that has been better studied in recent years, mainly because of the increased survival of cancer patients. In the cancer population, renal dysfunction persists in 23% of ICU survivors after discharge (30), 12.9% become dependent on RRT (31), and 13% progress to chronic kidney disease in childhood or adulthood (32), with CKD severity being proportional to AKI severity (33). These factors have repercussions of limiting the available first-line chemotherapy regimens or the optimized doses that can be administered.

In a prospective study of 200 patients with high-grade hematological malignancy without previous or first-line treatment (non-Hodgkin’s lymphoma, acute myeloid leukemia, acute lymphoblastic leukemia and Hodgkin’s disease) admitted to the ICU, with an incidence of AKI of 68%, the percentage of patients with complete hematological remission at 6 months was greater in patients without AKI than in those with AKI (68.3% vs. 39.1%), which is attributable to 14.6% of patients requiring a change in their chemotherapy regimen due to renal dysfunction. Among those who required a change in cancer treatment, 20% required a change in the dose of cytarabine, 15% required a change in the dose of cyclophosphamide, 5% required a change in the dose of oxaliplatin, and 50% required a suspension of methotrexate. Even among those who maintained their treatment regimen with full doses (85.4%), mortality was greater in the AKI group, suggesting that AKI per se is a risk factor for a worse cancer prognosis in this population (34), not only because of changes in drug pharmacokinetics but also because of other conditions attributable to the persistence of renal dysfunction, such as frailty, chronic inflammation and immunological alterations.

Although the classical classification of AKI into prerenal, intrinsic, and postrenal causes remains relevant from a pathophysiological standpoint, it is the intrinsic etiologies—such as acute tubular necrosis, interstitial nephritis, and glomerular injury—that most often persist beyond the acute phase and interfere significantly with cancer treatment planning. Prerenal causes, when promptly recognized and managed through adequate volume resuscitation, tend to resolve without long-term sequelae. Similarly, postrenal causes, such as obstructive uropathy, are frequently reversible with surgical or procedural interventions. In contrast, intrinsic AKI is more likely to evolve into persistent renal dysfunction or chronic kidney disease, thus imposing sustained limitations on chemotherapy choices and dosing.

Renal involvement in patients with malignancies can result not only from direct tumor effects or anticancer therapies (35, 36) but also from paraprotein-mediated mechanisms, which may be subtle and easily overlooked. Among these, monoclonal gammopathy of renal significance (MGRS) has emerged as a clinically relevant entity, characterized by the production of monoclonal immunoglobulins by small B-cell or plasma cell clones that do not meet criteria for multiple myeloma or lymphoma. These immunoglobulins may induce a range of renal lesions, including myeloma cast nephropathy, light chain deposition disease, and proliferative glomerulonephritis with monoclonal immunoglobulin deposits (PGNMID). Clinically, these disorders may present as acute kidney injury (AKI), subnephrotic or nephrotic proteinuria, or progressive chronic kidney disease, often in the absence of hematologic symptoms. Given this heterogeneity, kidney biopsy is essential for establishing an accurate diagnosis, especially in oncologic patients with unexplained renal dysfunction or disproportionate urinary abnormalities. Beyond clarifying etiology, histopathology guides therapeutic decisions, including the early initiation of clone-directed therapy in selected MGRS cases, thereby preventing further renal damage.

Renal toxicity remains a major limiting factor in the management of cancer patients, especially those requiring ICU care (37, 38). Several chemotherapeutic agents, including cisplatin, methotrexate, and alkylating agents such as ifosfamide and cyclophosphamide, are known to carry substantial nephrotoxic potential. Cisplatin is especially notable for its propensity to induce acute kidney injury (AKI), warranting preventive strategies such as aggressive hydration and diuresis. High-dose methotrexate may cause AKI through the precipitation of metabolites like 7-hydroxy-methotrexate within the renal tubules; in these cases, urine alkalinization, folinic acid rescue, and careful monitoring of serum levels are essential to minimize renal damage. Ifosfamide, in particular, has been associated with acute tubular dysfunction and Fanconi syndrome, further complicating patient management. These toxicities not only worsen clinical outcomes but can also delay or prevent the continuation of potentially life-prolonging cancer therapies (1).

Therefore, we now know that AKI is not a condition limited to the course of a critical illness but also involves the persistence of renal dysfunction after discharge from the ICU (39–42), a reality that is also present in the oncohematological population. Furthermore, the high mortality at 6 months in this population is apparently not attributable only to the need for dose adjustment of chemotherapeutic drugs.

## Conclusion

Despite the significant improvement in survival among critically ill cancer patients over the past decades, the long-term consequences of intensive care, particularly those encompassed by post-intensive care syndrome (PICS), have a growing impact on therapeutic decision-making in oncology.

In patients with aggressive hematologic malignancies, age over 65 years and persistent liver dysfunction have been identified as key barriers to maintaining optimal chemotherapy regimens. In patients with solid tumors, treatment continuation depends heavily on functional recovery, particularly performance status, and is influenced by tumor location (gastrointestinal and pulmonary sites being associated with worse outcomes).

Among all PICS-related complications, persistent renal dysfunction after AKI deserves special attention. It limits the use of first-line chemotherapeutic agents, requires frequent dose adjustments, and may reflect underlying systemic dysfunctions—such as chronic inflammation and immune impairment—that go beyond renal pharmacokinetics. The evidence suggests that AKI is not merely an acute event, but a turning point in the cancer trajectory of these patients.

Therefore, we propose the following actions:

- Implement structured post-ICU follow-up protocols for cancer patients, with emphasis on renal function monitoring and early detection of organ dysfunctions.
- Develop oncology-specific nephroprotective strategies during ICU stays, especially in patients receiving nephrotoxic therapies.
- Promote prospective studies focused on the relationship between AKI recovery, frailty, and chemotherapy tolerance in hematologic and solid tumors.

Finally, future research should aim to integrate the evaluation of PICS domains—especially physical and renal impairment—into cancer care pathways. Understanding how these factors influence long-term outcomes is essential to guide therapeutic decisions, personalize treatment plans, and improve survival with quality of life.

## Data Availability

The original contributions presented in the study are included in the article/supplementary material. Further inquiries can be directed to the corresponding author.
